# A Community-Informed Maternal and Infant Health Needs Assessment in Alabama

**DOI:** 10.1007/s10995-024-03988-2

**Published:** 2024-09-30

**Authors:** Holly Horan, Emily Locke, Lilanta Joy Bradley

**Affiliations:** 1 Department of Obstetrics and Gynecology, University of Alabama at Birmingham, 176F, 10360E, 619 19th ST South, Birmingham, AL 35249 USA; 2 Department of Anthropology, University of Alabama, 350 Marrs Spring Rd, Tuscaloosa, AL 35401 USA; 3 Department of Community Medicine and Population Health, University of Alabama, 850 Peter Bryce Boulevard, Tuscaloosa, AL 35401 USA

**Keywords:** Maternal health services, Needs assessment, Physicians, Patients, Alabama, Respectful maternity care

## Abstract

**Background:**

Maternal mortality is a global clinical and public health crisis. Researchers and leading organizations have highlighted the need for local partnerships to implement evidence-based strategies to mitigate poor outcomes. Alabama has the third highest maternal mortality rate in the nation. Research on the complexity of maternity mortality is strengthening, but poor outcomes in Alabama persist and there is limited data highlighting the perspectives of those on the frontlines of providing and receiving care.

**Purpose:**

We conducted a qualitative, statewide, community-informed, maternal and infant health research assessment with physicians, providers, professionals, and birthing persons to identify challenges and solutions to addressing the states’ poor perinatal health outcomes.

**Methods:**

Data were collected using a four-phase, research design that included semi-structured interviews, focus groups, one state-wide data sharing event, and five regional data sharing events. Data were collected between January 2020 and October 2021. The data were analyzed using consensus coding and thematic analysis.

**Main Findings:**

Fifty-nine (N = 59) individuals participated. Three themes emerged: 1) “They were making me feel so overlooked.”: A disconnect between perinatal healthcare services and patient needs; 2) “That shouldn’t be something you have to ask for.”: Limitations to providing respectful perinatal healthcare; and 3) “If they work together, they can have all the tools they need.”: Building a case for collaborative care.

**Conclusions:**

Participants advocated for a collaborative perinatal healthcare model that focuses on the provision of respectful, quality perinatal healthcare. Our approach can be applied across contexts and used to support the effective implementation of contextually relevant maternity care practices.

**Supplementary Information:**

The online version contains supplementary material available at 10.1007/s10995-024-03988-2.

## Significance

Evidence-based perinatal healthcare practices facilitate maternal and infant wellbeing; however, they are often underutilized. Community-based assessments are critical to understanding the landscape of perinatal healthcare and the priorities of those most affected within a specific context. These perspectives are critical to the development of effective evidence-based interventions. This study is the first qualitative assessment of maternal and infant healthcare research needs assessment in Alabama. We describe how perceptions and practices of care, based on the experiences of practitioners and birthing persons, are driven by health system limitations. Creating a culture of respect and expanding access to perinatal care options are critical to improving outcomes.

**Supplementary Information:**

The online version contains supplementary material available at 10.1007/s10995-024-03988-2.

## Introduction

Globally, maternal mortality is “unacceptably high”, totaling 800 preventable deaths per day, with a maternal death occurring every 2 minutes (MacDorman et al., 2016; World Health Organization (WHO), 2023). *Maternal mortality* is defined as the death of a person 42 days after their pregnancy has ended (Centers for Disease Control and Prevention (CDC), 2023a). The maternal mortality rate (MMR) is a national measure of equity and quality in perinatal healthcare (Creanga et al., 2014). Leading causes of maternal death include mental health, cardiovascular conditions, hemorrhage, thrombotic embolism, and hypertensive disorders (CDC, 2023a). More than 50% of maternal deaths occur one week or more postpartum and 80% of pregnancy-related mortality is preventable (Creanga et al., 2014; CDC, 2022). Between 2000 and 2020, the global MMR decreased by 34%, with notable slows in progress beginning in 2015 (WHO, 2023). Maternal mortality, globally, is a clinical and public health crisis, however, pinpointing effective interventions remains challenging.

In the United States (US), the MMR doubled between 1990 and 2013, with a current rate of 32.9/100,000 live births (CDC, 2023a). This rate parallels a 200% increase in severe maternal morbidity (SMM) rates, defined by the CDC as “unexpected outcomes of labor and delivery that can result in significant short- or long-term health consequences” (CDC, 2024; Creanga et al., 2014). These outcomes are associated with demographic and social determinants. Black birthing people are three times more likely to die after a pregnancy has ended when compared to White birthing people (Hoyert, 2023). Other factors associated with maternal mortality in the US include socioeconomic status, a lack of access to healthcare, and geography (CDC, 2022; Kozhimannil et al., 2017). Researchers and federal authorities have highlighted the need for local partnerships to implement evidence-based strategies to mitigate inequities in perinatal healthcare. The CDC has indicated that epidemiological data should be accompanied by complementary (e.g., qualitative) data to elicit the lived experience of communities and to assess the extent to which evidence-based strategies are desirable and feasible within a local context (CDC, 2023b).

Alabama, a southern US state, has the third highest MMR nationally and high rates of infant mortality and preterm birth among other poor maternal and child outcomes (Alabama Department of Public Health (ADPH)), 2022). Research on perinatal health in Alabama is growing, however, poor outcomes persist and there is limited data highlighting the perspectives of those on the frontlines of providing and receiving care. We conducted a qualitative, statewide, community-informed, maternal and infant health needs assessment to elicit the experiences of physicians, providers, professionals, and birthing persons working and living in Alabama—with a focus on barriers and facilitators to improving perinatal healthcare and outcomes. Our goal is to uplift and utilize the perspectives of participants to support the improvement of perinatal healthcare in our state and to encourage the use of qualitative data to create healthcare service delivery interventions that are based on the perspectives of those who provide and receive care.

## Methods

### Research Design

Data were collected using a four-phase research design that included semi-structured interviews with perinatal physicians, providers (i.e., midwives, advanced practice clinicians) and professionals (i.e., public health professionals) from multiple practices and institutions, focus groups with birth parents, a state-wide virtual data sharing event, and in-person, regional data sharing events. Data were collected between January 2020–October 2021 across Alabama’s five perinatal regions. Verbal, informed consent was collected from all participants. To protect confidentiality, participants were assigned a unique code (e.g., “MIH01”) upon consent. These codes were changed to pseudonyms by the research team. The exact professions of the perinatal providers and professionals by region are not specified to protect confidentiality. The Institutional Review Board at University of Alabama approved this study in December of 2019.

### Semi-Structured Interviews

Recruitment began in January 2020 using convenience, snowball sampling. Physicians, providers and professionals (hereafter, P^3^) who had worked in Alabama for at least five years and were 18 years or older were eligible to participate. The research team created an excel spreadsheet with 30 potential P^3^ participants from all five perinatal regions. Prospective participants were emailed or called by a member of the research team. Interviews were conducted from January to December 2020, shifting from in-person to Zoom due to the COVID-19 pandemic. A semi-structured guide was created to ensure consistency in data collection. Interview guide questions included demographic information (age, training, occupation, years in practice, etc.), professional experience with perinatal healthcare in Alabama, perspectives on poor perinatal health outcomes and existing programs to address these issues, and ideas about how to improve perinatal healthcare. Interviews were audio recorded, transcribed verbatim, and coded using NVivo–QSR International^®^. Participants received a meal and $15 virtual gift-card for participating.

### Focus Groups

Recruitment for focus groups began in March 2021 using convenience, snowball sampling. The research team collaborated with interview participants to distribute a virtual flyer about the focus groups via social media, email, and by phone. Interested participants emailed the primary investigator of the research team, who collected verbal, informed consent, and screened prospective participants. Participants were eligible if they had given birth in Alabama within the last 5 years and were 18 years or older. A focus group occurred in each of the five perinatal regions. Focus groups lasted 90 min and were conducted via Zoom between March and May 2021. A semi-structured focus group guide was created to ensure consistency in data collection. The guide included four questions that were similar to the interview guide and designed to elicit what participants believed were the greatest maternal and infant health issues in Alabama and solutions to address these issues. Participants completed a post-focus group survey online collecting their age, ethnicity, and county of residence. Each focus group was audio and video recorded, transcribed verbatim, and coded using NVivo-QSR International^®^. Focus group participants each received a $25 door-dash gift-card and a $30 gift-card.

### Data Sharing Events

After conducting preliminary analysis, the research team hosted data sharing events with participants at the state and regional level. The first data sharing event included participants from the entire state-wide sample and was hosted on Zoom in August 2021. This event served as a form of member checking where the researchers shared preliminary results with participants and elicited their feedback on how to move forward with analysis (Maxwell, 2012). Preliminary findings were shared using PowerPoint and included demographic data, an overview of the data collection process, and preliminary themes from the data. The event concluded with an open dialogue session with participants. Later in fall 2021, the research team held one, in-person local data sharing event in each perinatal region. These events had limited attendance due to the COVID19 pandemic and schedule conflicts. The state-wide presentation was modified for each of the regional data sharing sessions with data specific to each region. Participants were encouraged to share their interpretation of the findings. Member checking, at the state and region level, is important because it is a method of validating the credibility of the data and the results (Merriam & Tisdale, 2015). The research team took detailed notes at each event.

### Data Analysis

The research team transcribed and coded the interview data from January–March 2021. We used a consensus coding approach, which allows for a diverse range of codes to be identified in the narrative data because two or more coders are conducting the analysis. This approach is critical, as researcher positionality is known to influence which themes are identified and prioritized and which were overlooked (Maxwell, 2012). The research team independently coded the interview data using an inductive approach. The team met to discuss and deliberate their preliminary codes and reached consensus on five overarching codes and 16 subcodes. The research team then re-coded the entire dataset using a codebook that consisted of these codes and subcodes. Then the research team met again to review their coding and to compare similarities and difference across the sets of coded data using the comparative method (Bingham, 2023). Finally, the research team engaged in thematic analysis by grouping conceptually similar codes together and operationalizing them using key quotes and concepts from the participant narratives (Braun & Clarke, 2006, 2019).

Data analysis for the focus groups occurred from June–September 2021. The research team began by reviewing the focus group recordings. We used the same analytical approach for focus groups and the interview data. After completing inductive, consensus coding, the team met and created a codebook that included four primary codes. Then we recoded the entire focus group dataset, met to compare the coding results, and engaged in thematic analysis. The research team cross-examined the interview and focus group codes and preliminary themes and identified that there were three overarching themes that cross-cut the entire data set. Member checking during the data sharing events assisted with validating and modifying the final results.

## Results

Fifty-nine (*N* = 59) individuals participated in this study (see Tables 1 and 2). All birthing persons identified as mothers. Of the 59 participants, 26 attended the state-level virtual data sharing event and 12 attended the local data sharing events. All participant groups were represented at the data sharing events. Three themes were identified: (1) “They were making me feel so overlooked.”: A disconnect between perinatal healthcare services and patient needs; (2) “That shouldn’t be something you have to ask for.”: Limitations to providing respectful perinatal healthcare; and (3) “If they work together, they can have all the tools they need.”: Building a case for collaborative perinatal care.

**Table 1:** Participant sample by data collection measure

Data Collection Tool	*n*
Interviews (P^3^)	25
Focus Groups (mothers)	34
**Total**	59
Data Sharing Events	*n*
State-wide	26
Region I	2
Region II	2
Region III	2
Region IV	3
Region V	3

**Table 2:** Demographic characteristics of participants

	*P* ^3^		Birth Parents		Full Sample	
	*n*	%	*n*	%	*n*	%
Perinatal Region						
Region I	6	24	7	21	13	22
Region II	6	24	5	15	11	19
Region III	4	16	6	18	10	17
Region IV	4	16	3	8	7	12
Region V	5	20	13	38	18	30
Race/Ethnicity						
Black	4	16	9	26	13	22
Latinx/Hispanic White	1 20	4 80	1 24	4 70	2 44	3 75
Age						
Median	39.5		33		35	
Range	28–64		20–40		20–64	

*Theme 1: “They were making me feel so overlooked.”*: A disconnect between perinatal healthcare services and patient needs

Participants explained that there was a lack of meaningful communication and relationship building between patients, their healthcare providers, and other care team members. They described how this lack of rapport reduces the efficacy of perinatal healthcare and health outcomes. For P^3^, barriers to building rapport during a clinical encounter included limited time and high patient-loads, causing them to “hit the burn-out mode so fast.” Erin, a perinatal health professional, described how patients notice these barriers: “…they’re starting to realize it’s not okay when they [the patient] come in for an OB appointment and the doctor stands at the door with their hand on the door knob the whole time.” Mothers discussed the potential danger caused by a lack of rapport between patients and clinical staff, and how they, as patients, were not feeling heard. Ava, a mother of one, describes her experience when she presented with symptoms of postpartum eclampsia: “…they [the clinical staff] were making me feel…so overlooked. When I see those stories about Black women going to the hospital and not being heard and dying it’s like ‘this could have happened to me’.”

All mothers, irrespective of race, recognized that a lack of effective communication and relationship building could be a matter of life and death. They shared related experiences of discrimination based on gender and socioeconomic status, agreeing that this led to medical distrust and diminished rapport with clinical staff. All participants acknowledged how this causes perinatal health issues to “slip through the cracks.” Many mothers felt that they did “not know what to expect” when it came to birth and postpartum, even after attending their prenatal care appointments. They also expressed that they were “afraid” to question their providers.

Participants reflected on the tension between patient and provider roles when it came to establishing rapport within the context of perinatal care. Casey, a mother and perinatal healthcare professional explained: “…you want to be a good mom, so you want to be the best patient.” Based on her professional experience, Casey explained that practice constraints, such as limited time during prenatal care visits, made it difficult to provide “…all the options so moms can pick what’s best for themselves and their family” and that this limitation contributed to the reality that patients felt unheard.

*Theme 2: “That shouldn’t be something you have to ask for.”: Limitations to providing respectful perinatal healthcare*

Participants described how rapport building was connected to the provision of respectful perinatal healthcare. While many of the participating mothers recognized that clinical providers are experts, they felt that, during healthcare visits, clinical expertise overshadowed patients’ knowledge about their bodies and needs. This issue was critical for participants who identified as Black and for those who experienced emergent medical events. Grace, a social worker and perinatal mental health specialist reflected on the perceived disrespect patients experience:

“…I’ve never met so many Black mothers and mothers in general who can’t get access to care and a doctor to listen to them…Often mothers are stuck with who is available to care for them in the service area…or…they don’t know better, and they stay with their provider even if they have their doubts.”

Mona, a mother of two, elaborated from the patient perspective: “I did not have a lot of options for OBs, and I could not stand my provider for my first pregnancy. When I found out that my doctor…was not on call the day I gave birth, I cried in happiness.”

Ashley, a pregnant mother with two children, also described how perceived disrespect can result in birth trauma—leaving the mother feeling like “…the baby is the candy, and the mother is just the wrapper.” Casey, a mother and perinatal healthcare professional, used her own lived experience to illustrate this scenario after a medically-indicated cesarean birth:

“I was feeling vulnerable and didn’t know to ask for things…Now I know I could have asked for the baby to stay with me, I could have asked for skin-to-skin, but that shouldn’t be something you have to ask for, it should be something that is given to you.”

Hannah, a mother of three, describes how she approached her OB after her traumatic third birth to learn more about what happened to her:

“My OB said: ‘We see this stuff all the time and we just don’t think about it…’ Then my OB sat down with me, brought my file in and told me everything that happened. After that, everything made more sense.”

These experiences highlight how effective communication, rapport building, and respectful care can be critical to a patient’s wellbeing during pregnancy, labor and birth, and postpartum.

*Theme 3: “If they work together*,* they can have all the tools they need.”: Building a case for collaborative perinatal healthcare*

Participants emphasized that high quality perinatal healthcare is essential and should be available to *all* pregnant people. They also recognized that there is limited capacity to realize this in Alabama. Participants identified many barriers to providing quality care, including health insurance, transportation, poverty, broadband, and the closing of rural healthcare facilities. Physicians, providers and professionals (P^3^) described how systems level gaps made providing collaborative care challenging because many counties and cities have “…absolutely no clinics or infrastructure to sustain healthcare providers.” They also explained that healthcare access challenges are due to “…a lack of access to healthcare and insurance coverage.”

Many participants identified the need for more racially diverse healthcare professionals. In particular, they indicated that racial congruence is a priority for patients of color, under the condition that this racially concordant provider values building rapport and providing unbiased, respectful care to their patients. Dana, a mother of two, described the importance of these conditions when she recounted how she found her obstetric provider:

“…being African-American, most of us feel more comfortable with someone that looks like us and she [Dana’s friend] initially started with someone that looked like us and it was terrible…It was like judgment…I helped her [Dana’s friend] find a new provider and I fell in love with her [Dana’s friend’s provider]. I knew when I had a baby, that I would want to go to her…she wasn’t Black and it does not bother me. But…you know going into it we think that somebody that looks like us would understand, or you know, just be…an unbiased provider.”

Participants also elaborated on the need to expand access and availability of perinatal healthcare options, including midwifery care and doula support. Ruth, a perinatal healthcare professional, stated:

“I would like to see expanded utilization and state support for scaling up perinatal care options, so that they are accessible… This includes more home birth, birth centers, more collaborative care, and group prenatal care.”

Amelia, a mother of one, emphasized the importance of doula support, stating: “…we definitely need doula care for all women who want it…they should always be able to pull the ‘doula card’ even if the person in labor and doesn’t know they need it until right in that moment.”

Participants recognized that the implementation of respectful, collaborative perinatal healthcare would require protected time and training for P^3^. Many agreed that medical students and residents should receive training about community birth options (i.e., a birth center or home setting). Olivia, a clinical provider and support professional, described: “[T]hey don’t know what it [community perinatal healthcare] looks like because they haven’t been trained in it.” Participants agreed that this training could promote a culture of mutual respect, collaboration, and evidence-based practice and that this could increase the provision of quality, respectful care for patients. Beth, a clinical staff member and support professional describes the importance of collaboration and respecting each professionals’ skill set, stating: “OBs …their skills are also tools [like midwives, nurses, and doulas] and we can move seamlessly between all of those. It’s just all about…bridge building. If they work together, they can have all the tools they need.”

## Discussion

Participants emphasized that improving maternal healthcare outcomes in Alabama requires fostering a culture of *respectful perinatal healthcare*. A growing body of research on respectful maternity care (RMC) emphasizes that every perinatal person “…deserves respectful and responsive care during pregnancy and delivery” (CDC, 2023b; Mohamoud et al., 2023; Vedam et al., 2019). At the interpersonal level, respectful care begins with better communication, a central component of RMC (Mohamoud et al., 2023). However, approximately 45% of perinatal patients report holding back questions or sharing concerns with their maternity care provider (CDC, 2023b). This occurs because patients feel that their provider is rushed or patients lack the confidence (and trust) to discuss concerns with them (2023b)—data that corroborates the perspectives of the study participants. Poor provider-patient communication is associated with an increased risk of negative maternal health outcomes (CDC, 2023b; Vedam et al., 2019). Quality provider communication with patients is characterized by a clear agenda, active listening, attention to non-verbal cues, asking for the patient’s perspective, and using empathetic language (Dugdale et al., 1999; Morton & Simkin, 2019). This has been associated with accurate and timely diagnosis and treatment of complications as well as increased patient satisfaction and improved adherence to medical counseling (American College of Obstetricians and Gynecologists, 2014).

Disrespectful care, intentional or unintentional, is linked to mistreatment and causes patients to feel ignored, punished, unprotected, threatened, or neglected by their care providers (Vedam et al., 2019). These experiences disproportionately affect racially diverse perinatal persons and patients who are uninsured or receive public insurance (e.g., Medicaid) (Mohamoud et al., 2023). Participants indicated the need for a more racially diverse workforce that is comprised of the full range of evidence-based perinatal care providers. Racial concordance has been shown to be helpful in improving infant health outcomes with research suggesting that this effect could translate to maternity care (Greenwood et al., 2020). Racially concordant care should be prioritized; however, trust and safety must also be established with the patient to realize the full benefits of concordant care (O’Rourke et al., 2022). National organizations also strongly encourage healthcare systems to hire and retain a diverse workforce and identified that midwives and doulas are excellent facilitators of communication and support for patients (The White House, 2022; Mohamoud et al., 2023).

Participant perspectives provide critical insight about how to provide respectful care in Alabama. A recent systematic review highlighted that the terms “teamwork” and “communication” among physicians, providers, other staff, and patients are not explicitly included in measures of RMC (AHRQ, 2024). Interestingly, these concepts were explicitly voiced by our study participants as critical elements of respectful care, in addition to patient and provider communication which is part of the RMC model. Participants also provided insight into training gaps that need to be filled to facilitate the provision of respectful perinatal care. While there is a wealth of trainings related to bias, racism, and other forms of discrimination—participant narratives indicated a need for more formal training in topics and skills such as motivational interviewing, trauma-responsive care, and community/patient engagement. Participant recommendations also identified the need for cross-disciplinary training and collaboration with clinical and community-based clinicians and professionals, with the goal of having a greater understanding and mutual respect for these professionals who have complementary scopes of practice.

## Conclusion

The provision of quality, respectful maternity care includes partnerships with communities to increase awareness and promote health equity (Mohamoud et al., 2023). This is critical, given the national and international maternal mortality crisis. Our qualitative, statewide, community-informed, maternal and infant health needs assessment was designed to uplift the voices of individuals on the frontlines of perinatal healthcare (physicians, providers, professionals, and birthing persons) in Alabama. Participants advocated for a collaborative, respectful model of perinatal healthcare by identifying essential issues that affect the provision of quality perinatal healthcare in Alabama and elsewhere: poor communication, a lack of rapport building, and disrespectful care. Our approach can be applied across contexts and used to support the effective implementation of respectful maternity care practices that respond to the needs and perspectives of local healthcare providers, professionals, and patients.

## Electronic Supplementary Material

Below is the link to the electronic supplementary material.

Supplementary Material 1

## Data Availability

Data and supplemental materials will be made available upon request.
