# Correction: A Community-Informed Maternal and Infant Health Needs Assessment in Alabama

**DOI:** 10.1007/s10995-024-04013-2

**Published:** 2024-10-22

**Authors:** Holly Horan, Emily Locke, Lilanta Joy Bradley

**Affiliations:** 1 Department of Obstetrics and Gynecology, University of Alabama at Birmingham, 176F, 10360E, 619 19th ST South, Birmingham, AL 35249 USA; 2 Department of Anthropology, University of Alabama, 350 Marrs Spring Rd, Tuscaloosa, AL 35401 USA; 3 Department of Community Medicine and Population Health, University of Alabama, 850 Peter Bryce Boulevard, Tuscaloosa, AL 35401 USA

**Correction to: Maternal and Child Health Journal**

10.1007/s10995-024-03988-2

The original version of the article unfortunately contained an error in text.

In introduction section, the citation “Centers for Disease Control (CDC), 2023a” should have been “Centers for Disease Control and Prevention (CDC), 2023a”.

In Data Analysis section, the word “occurred” must be deleted from the first sentence and it should read as “The research team transcribed and coded the interview data from January–March 2021.”

In Results section, the part of the text “If they work together, they can have all the tools they need.” was cut-off in the last sentence under Theme 3. So, the last sentence should have read as “OBs…their skills are also tools [like midwives, nurses, and doulas] and we can move seamlessly between all of those. It’s just all about…bridge building. If they work together, they can have all the tools they need.”

The original article has been corrected.

