# Supplementary material for: A Community-Informed Maternal and Infant Health Needs Assessment in Alabama
Source: Matern Child Health J. 2024 Sep 30;28(11):1833–9. doi: 10.1007/s10995-024-03988-2 (PMC11534882; doi:10.1007/s10995-024-03988-2)
Supplement: Supplementary file 1 — Supplementary Material 1 [file 10995_2024_3988_MOESM1_ESM.docx]

**Background:** Maternal mortality is a global clinical and public health crisis. Researchers and leading organizations have highlighted the need for local partnerships to implement evidence-based strategies to mitigate poor outcomes. [Hidden] has the third highest maternal mortality rate. Research is increasing, but poor outcomes in [hidden] persist and there is limited data highlighting the perspectives of those on the frontlines of providing and receiving care.

**Purpose:** We conducted a qualitative, statewide, community-informed, maternal and infant health research assessment with physicians, providers, professionals, and birthing persons to identify challenges and solutions to addressing the states’ poor perinatal health outcomes.

**Methods:** Data were collected using a four-phase, research design that included semi-structured interviews, focus groups, one state-wide data sharing event, and five regional data sharing events. Data were collected between January 2020 and October 2021. The data were analyzed using consensus coding and thematic analysis.

**Main Findings:** Fifty-nine (N=59) individuals participated. Three themes emerged: 1) “They were making me feel so overlooked”: A disconnect between perinatal healthcare services and patient needs; 2) “That shouldn’t be something you have to ask for”: Limitations to providing respectful perinatal healthcare and 3) “If they work together, they can have all the tools they need.”: Building a case for collaborative care.

**Conclusions:** Participants advocated for a collaborative perinatal healthcare model that focuses on the provision of respectful, quality perinatal healthcare. Our approach can be applied across contexts and used to support the effective implementation contextually relevant maternity care practices.

**Significance**

Evidence-based perinatal healthcare practices facilitate maternal and infant wellbeing; however, they are often underutilized. Community-based assessments are critical to understanding the landscape of perinatal healthcare and the priorities of those most affected within a specific context. These perspectives are critical to the development of effective evidence-based interventions. This study is the first, qualitative assessment of maternal and infant healthcare research needs assessment in [hidden]. We describe how perceptions of care, for practitioners and birthing persons, are driven by limitations in the provision of perinatal healthcare. Creating a culture of respect and expanding access to perinatal care options are critical.

**Keywords:** Maternal health services, Needs assessment, Physicians, Patients, [Hidden]
